# Influence of Invisalign treatment with interproximal enamel reduction (IER) on bone volume for adult crowding: a retrospective three-dimensional cone beam computed tomography study

**DOI:** 10.1186/s12903-016-0281-1

**Published:** 2016-09-01

**Authors:** Andreas Hellak, Nicola Schmidt, Michael Schauseil, Steffen Stein, Thomas Drechsler, Heike Maria Korbmacher-Steiner

**Affiliations:** 1 Department of Orthodontics, University Hospital Giessen and Marburg, Campus Marburg, Georg-Voigt-Strasse 3, Marburg, 35039 Germany; 2 Private practice, Wiesbaden, Germany; 3 Abt. für Kieferorthopädie, UKGM Standort Marburg, Georg-Voigt-Strasse 3, 35039 Marburg, Germany

**Keywords:** Invisalign, Adult crowding, Interproximal enamel reduction (IER), Bone quantity, CBCT, Landmarks, Recessions

## Abstract

**Background:**

The aim of this study was to use three-dimensional datasets to identify associations between treatment for adult crowding using Invisalign and interproximal enamel reduction (IER) and changes in the bone volume.

**Methods:**

A total of 60 digital cone beam computed tomography (CBCT) scans from 30 patients (28 women, two men; 30 CBCTs pretreatment, 30 posttreatment) were examined retrospectively in order to record bone volume three-dimensionally before and after treatment. The patients’ average age was 36.03 ± 9.7 years. The data were collected and analyzed using the computer programs Mimics 15.0 and OsiriX. Differences in bone between T0 and T1 were analyzed with IBM SPSS 21.0 using the Wilcoxon test for paired samples.

**Results:**

Analysis of the orovestibular bone volume showed highly significant changes (bone change *P* <0.001) only in the mandible where more expansion of the dental arch was carried out using proclination or protrusion. The bone lamella was thinner buccally and thicker lingually. In general, bone increases in the oral direction were slightly greater than bone losses in the vestibular direction. No significant changes were detected in the maxilla (bone change *P* = 0.13). Significant vertical bone loss in the bone height was detected in both the maxilla and the mandible. The largest bone loss was observed in the vestibular direction in the mandible, at a high level of significance (*P* <0.001).

**Conclusions:**

Particularly in the mandible, therapeutic reduction of the vertical and sagittal bone volume shows that caution should be used in the treatment of tertiary crowding with proclination and expansion. The cortical walls appear to represent the limits for orthodontic tooth movement, at least in adult female patients.

## Background

Treatment for adult patients using almost invisible appliances such as aligners is becoming increasingly important [1]. In addition to harmonizing the dental malpositioning, with the consequent improvement of the patient’s aesthetic appearance, simultaneous improvement of dental health is also desirable [2].

However, orthodontic treatment for adult crowding has been associated in many reports with recessions and periodontal destruction in the area of the anterior teeth [3–6]. It is known that dental movements cause quantitative changes in the alveolar bone, with osteogenesis and osteoclasis [7–9]. As a result of the two-dimensional imaging that has been conventional to date, quantification of the pretherapeutic and posttherapeutic bone situation is only possible to a limited extent [10]. It is only through modern three-dimensional imaging procedures such as cone beam computed tomography (CBCT) that three-dimensional analysis of bone structures has become possible [11].

There have been no reports in the literature on changes in the bone volume relative to treatment with aligners. The aim of the present study was therefore to investigate whether and to what extent orthodontic treatment with Invisalign aligners leads to changes in the bone volume. Specifically, this raises the following questions:

- How does the bone volume change in the orovestibular direction as a result of Invisalign treatment?
- What changes in the bone height do occur?
- Are there differences in the ways in which the maxilla and mandible respond?
- Does the movement pattern have any effect on bone transformation processes?

## Methods

Two CBCT scans (pretherapeutic and posttherapeutic) from a total of 30 patients (28 women, two men) were examined retrospectively. The patients’ average age was 36.03 ± 9.7 years. A total of 920 measurements were carried out for orovestibular changes and 480 measurements for bone height. The use of the data was approved by the ethics committee of Marburg University Hospital (ref. no. 34/15).

The following inclusion criteria were used:

- Presence of adult anterior crowding capable of being harmonized using conservative orthodontic space-gaining measures such as protrusion, proclination, expansion and interproximal enamel reduction (IER)
- Permanent dentition
- Successfully completed treatment with Invisalign aligners
- Availability of one CBCT scan each from before and after treatment

The following parameters represented exclusion criteria:

- Extraction of anterior teeth during the course of treatment
- Prosthetic treatment
- Skeletal anomalies
- General medical findings relevant to bone metabolism (e.g., osteoporosis, dysostosis, etc.)
- Periodontal disease and previous periodontal surgery procedures

All of the images were taken with a KaVo 3D eXam CBCT system (KaVo Dental Ltd., Biberach an der Riss, Germany) using a scan with 360° revolution, 26,9 s duration (X-ray source voltage: 120kVp; X-ray source current: 5 mA) and 0.25 mm voxel size. The datasets were collected and evaluated using Mimics 15.0 (Materialise, Leuven, Belgium) with Microsoft Windows 7 and OsiriX (Pixmeo, Bernex, Switzerland) with an Apple OS X operating system.

All of the patients had provided written consent to the use of their data in the study (in accordance with the Helsinki Declaration). The datasets were all analyzed on a semiblinded basis.

### Measurement of the orovestibular bone volume

Bone thickness was measured orally and vestibularly at two levels (at the apex and mid of the root length) in the anterior tooth areas of the maxilla and mandible.
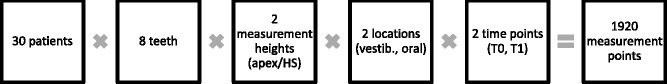


The upper and lower threshold values for the best possible depiction of the skeletal components of the skull were established using the “thresholding” function in Mimics 15.0 (Materialise, Leuven, Belgium).

**Fig. 1:**
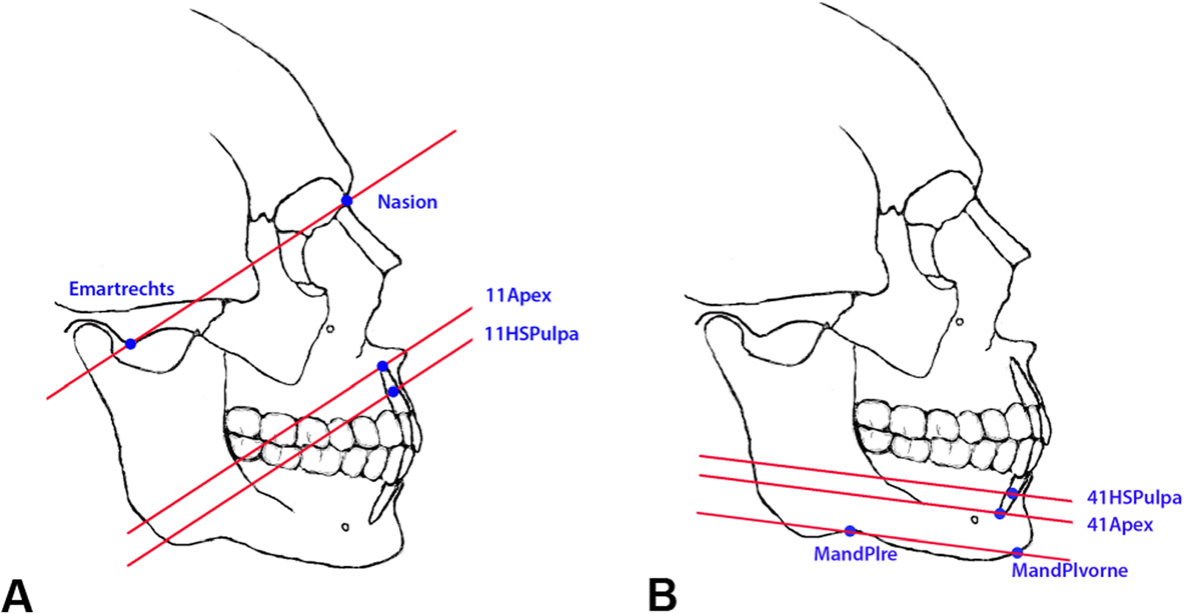
**a+b** Two-dimensional illustration of the maxillary and mandibular reference plane. **a** Two-dimensional illustration of the maxillary reference plane, with the reference points “Emartrechts” and “Nasion” and the parallel measurement planes at the level of the apex and center of the root, for tooth 11. **b** Two-dimensional illustration of the mandibular reference plane, with the reference points “MandPlre” and “MandPlvorne” and the measurement planes at the level of the apex and center of the root, for tooth 41

**Fig. 2:**
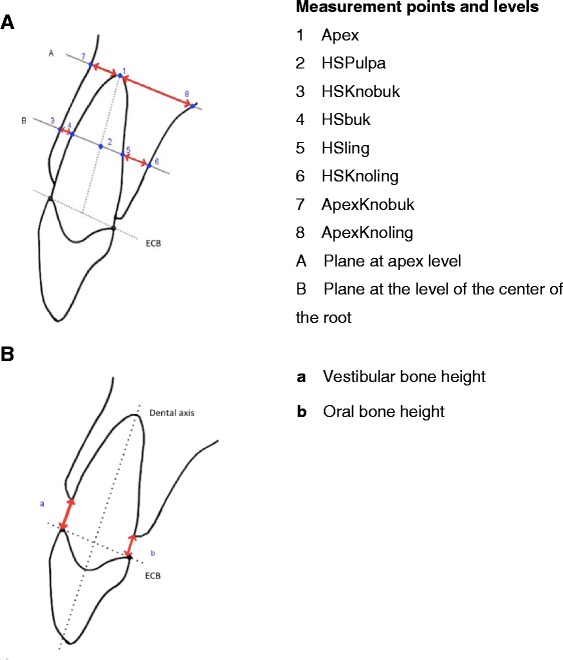
**a+b** Two-dimensional diagram showing the measurement points. **a** Sketch, Tooth 11 as an example. *ECB* enamel–cement boundary. Two-dimensional diagram showing the measurement points and distances for measuring bone height. **b** Sketch, Tooth 11 as an example. *ECB* enamel–cement boundary

To be able to carry out the measurements independently of the tooth position, reference planes first had to be created using osseous structures. The reference planes in the maxilla and mandible were created using clearly defined anatomic landmarks. Table 1 and Fig. 1 show the points used to establish the reference planes in the maxilla and mandible. Clearly defined reference points produced the distances shown in Fig. 2a, which were always measured vertically to the alveolar process and parallel to the previously established reference plane in the maxilla or mandible. The following measurement distances resulted:

- Apex–ApexKnoBuk: bone from the apex to the labial boundary of the cortex
- Apex–ApexKnoLing: bone from the apex to the oral boundary of the cortex
- HSbuk–HSKnoBuk: bone from the buccal root contour to the labial boundary of the cortex at the level of the mid of the root length
- Hsling–HSKnoLing: bone from the lingual/palatine root contour to the oral boundary of the cortex at the level of the mid of the root length

**Table 1:** Reference points for establishing reference levels in the maxilla and mandible

Reference level	Analysis points	Positioning
Maxillary level	Emartrechts	Deepest point on the right articular tubercle
	Emartlinks	Deepest point on the left articular tubercle
	Nasion	Furthest anterior contour of the frontonasal suture
Mandibular level	MandPlre	Deepest point on the mandible in the area of the mandibular angle on the right
	MandPlli	Deepest point on the mandible in the area of the mandibular angle on the left
	MandPlvorne	Deepest point in the area of the ventral mandible

**Fig. 3:**
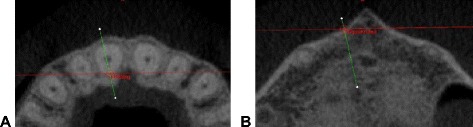
**a+b** Setting the reference points. Reference points at the intersection between the transverse and sagittal planes at the corresponding anatomic structure are shown using the example of tooth 11 at the axial plane. **a** “11Hsling” at the lingual root contour at the level of the center of the root. **b** “11ApexKnobuk” at the buccal bone contour at the level of the apex

Figure 3 shows an example of the way in which the measurement points were set. The red line shows the intersection point with the previously mounted plane. This made it possible for the measurement always to be carried out at the same level.

### Measurement of bone height

The distance from the enamel–cement boundary to the crestal alveolar bone on all four anterior teeth in the maxilla and mandible was measured using the OsiriX program (Pixmeo, Bernex, Switzerland).
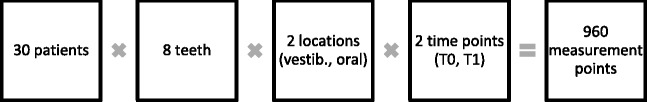


This method was adopted in a slightly modified form from the retrospective CBCT study by Kim et al. [12].

**Fig. 4:**
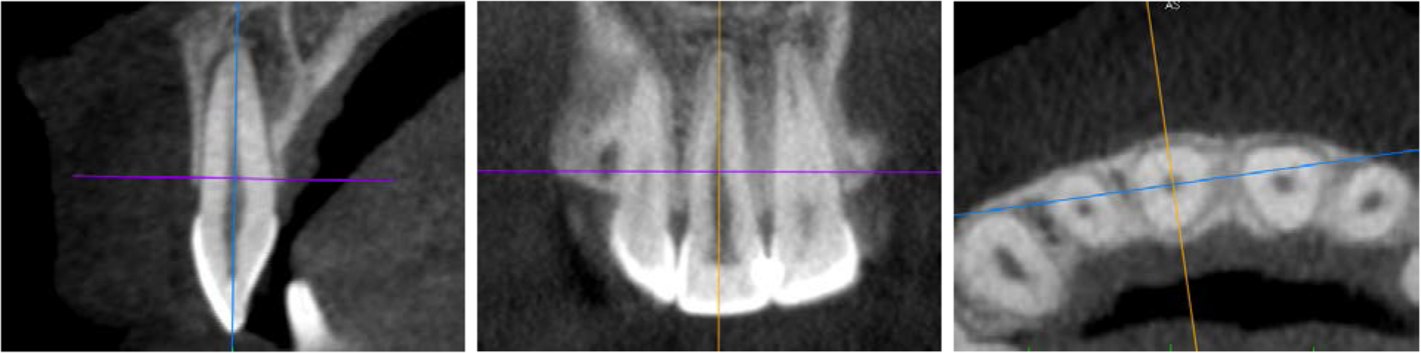
Measurement of the buccal and lingual bone height. Measurement of the bone height on tooth 11 was carried out parallel to the dental axis both at buccal root surface and at lingual root surface

The most coronal level of the alveolar bone was defined as the crestal bone ridge, independently of whether bone fenestrations were identifiable apical to that level. In the sagittal view, the distance from the crestal bone edge to the enamel–cement boundary was then measured using OsiriX’s measurement function. Measurements were carried out parallel to the dental axis both at the buccal root surface and at the lingual root surface (Figs. 2b and 4).

The following measurement distances resulted:

- Vestibular bone height (a): distance between the vestibular crestal bone edge and the enamel–cement boundary
- Oral bone height (b): distance between the oral crestal bone edge and the enamel–cement boundary

Figure 4 shows an example of the way in which the tooth is oriented in order to measure the bone height.

Statistical analyses were carried out using IBM SPSS for Mac, version 21.0 (IBM Corporation, Armonk, New York, USA). The intraoperator correlation for each examination was initially calculated. For further analysis, the normal distribution of the values was checked. Statistical comparison of the values was carried out using the Wilcoxon test. The significance level was set at *P* = 0.05.

## Results

A high level of correlation (*r* = 0.903) was noted using Kendall’s tau-b test for the examiner’s accuracy in measurements of the orovestibular bone thickness. The correlation was highly significant, at the *P* <0.001 level (two-sided). The Kolmogorov–Smirnov test did not show a normal distribution for measurements of vestibular and oral bone thickness. The Wilcoxon test was therefore used for statistical analysis of the bone thickness measurements (Table 2). Clear changes were only observed in the mandible. The sums of the mean differences showed bone losses in the maxilla and slight bone increases in the mandible. An increase in bone was evident at both levels for the mandibular measurement points, while bone loss was evident buccally.

**Table 2:** Comparison of differences in the sagittal bone thickness (in mm) between T1 and T0

	Position	Level		n	Min.	Max.	Mean	SD	Wilcoxon test T1–T0	
Maxilla	Buccal	Apex	T1–T0	120	−2.36	4,01	0,04	1,04	Z	−0.063 ^a^
									A. significance (two-sided)	*P = 0.950*
		HSPulpa (HEJ)	T1–T0	120	−1.19	1,36	0,02	0,41	Z	−0.965 ^b^
									A. significance (two-sided)	*P = 0.334*
	Oral	Apex	T1–T0	120	−3.87	2,66	−0.17	1,2	Z	−0.980 ^a^
									A. significance (two-sided)	*P = 0.327*
		HSPulpa (HEJ)	T1–T0	120	−1.94	1,34	−0.11	0,58	Z	−2.069 ^a^
									A. significance (two-sided)	*P = 0.039*
Mandible	Buccal	Apex	T1–T0	120	−2.59	3,6	−0.42	0,86	Z	−5.853 ^a^
									A. significance (two-sided)	*P <0.001*
		HSPulpa (HEJ)	T1–T0	120	−1.43	0,47	−0.17	0,25	Z	−6.646 ^a^
									A. significance (two-sided)	*P <0.001*
	Oral	Apex	T1–T0	120	−3.56	2,31	0,48	0,84	Z	−6.630 ^b^
									A. significance (two-sided)	*P <0.001*
		HSPulpa (HEJ)	T1–T0	120	−0.81	1,97	0,39	0,46	Z	−7.377 ^b^
									A. significance (two-sided)	*P <0.001*

Wilcoxon test* for statistical analysis

* Wilcoxon signed rank test

^a^ Based on positive ranks

^b^ Based on negative ranks

In the statistical analysis, the Wilcoxon test showed highly significant changes in the mandible (*P* ≤0.001). The results in the maxilla only showed significant differences in the palatine area at the level of the mid of the root length (HSPulpa; *P* ≤0.05) (Table 2).

### Bone height

A high level of intraoperator correlation was also observed for the measurements of bone height (Kendall’s tau-b: *r* = 0.779). The correlation was highly significant, at the *P* <0.001 level (two-sided).

The distance from the enamel–cement boundary to the alveolar crest was measured. Bone loss was indicated by positive values. For clarity, all values in the subsequent analysis of this part of the study were therefore multiplied by −1. This results in negative values for bone losses and positive values for bone increases on the vertical plane.

Significant vertical bone losses were identified in both the maxilla and the mandible (Table 3). This was true both for the buccal and also for the oral measurement points. Vertical bone loss was particularly marked buccally in the mandible (*P* <0.001). The lingual bone height in the mandible did not show any significant changes (*P* = 0.345).

**Table 3:** Comparison of differences in bone height (in mm) between T1 and T0

	Position		n	Min.	Max.	Mean	SD	Wilcoxon test T1–T0	
Maxilla	Buccal	T1–T0	111	−7.85	3,53	−0.24	1,15	Z	−3.058 ^a^
								A. significance (two-sided)	*P <0.01*
	Oral	T1–T0	119	−9.80	3,64	−0.57	1,86	Z	−3.287 ^a^
								A. significance (two-sided)	*P =0.001*
Mandible	Buccal	T1–T0	120	−9.99	6,4	−2.42	3,37	Z	−6.864 ^a^
								A. significance (two-sided)	*P <0.001*
	Oral	T1–T0	120	−6.74	4,65	−0.05	1,63	Z	−0.944 ^b^
								A. significance (two-sided)	*P =0.345*

Wilcoxon test* for statistical analysis

* Wilcoxon signed rank test

^a^ Based on positive ranks

^b^ Based on negative ranks

Tables were prepared to analyze the influence of the movement pattern on the way in which the bone reacted (Tables 4 and 5). When expansion of the dental arch was carried out using proclination/protrusion, the affected teeth were shown in red; in the absence of dental arch expansion, they were shown in green.

**Table 4:**
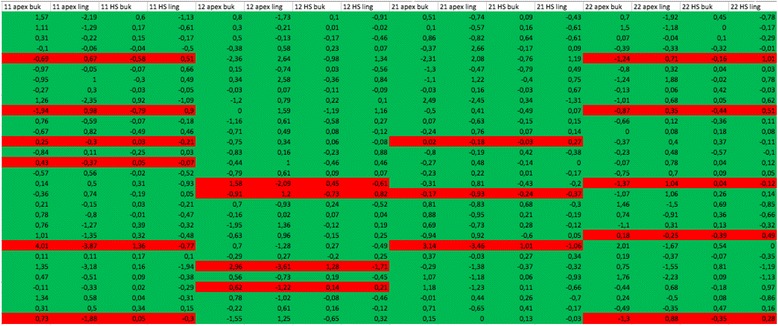
Frequency distribution for planned tooth movement in upper arch

Red: expansion of the dental arch in the maxilla using proclination/protrusion; green: proclination/protrusion not planned

**Table 5:**
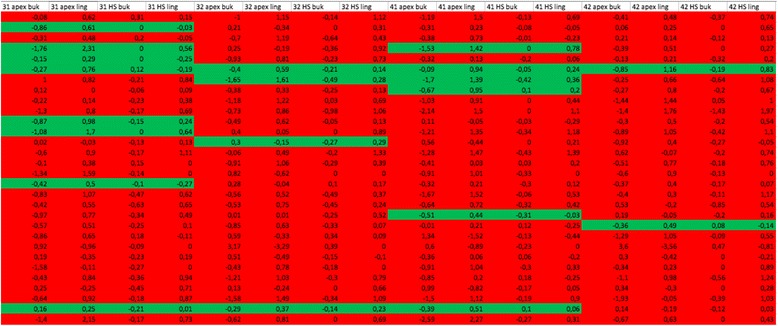
Frequency distribution for planned tooth movement in lower arch

Red: expansion of the dental arch in the mandible using proclination/protrusion; green: proclination/protrusion not planned

Tables 4 and 5 show that protrusion and proclination to expand the dental arch were selected more frequently as a treatment strategy in the area of the mandible. In the mandible 83.3 % of the teeth studied were moved labially, in comparison with only 15 % in the maxilla.

## Discussion

Highly significant changes in bone volume in the orovestibular direction were only found in the mandible. A reduction in the vestibular bone lamella and an increase in bone in the lingual area of the mandible were observed. This might possibly be explained by different movement patterns. Bone changes in the direction of the tooth movement. The present study only included patients with adult crowding. One possible noninvasive treatment for crowding involves expanding the dental arch labially in order to create space for normal positioning of the affected teeth [13]. This method of creating space was used much more often in the mandible. The results of reducing the bone thickness in the direction of the orthodontic tooth movement have been described previously in two-dimensional studies [14–16]. However, only limited visualization of the three-dimensional changes that take place in the bone is possible with two-dimensional images. To date, the specialist literature only includes some three-dimensional studies on this topic [17, 18]. In contrast to the present report, all of the patients included in these studies had been treated with multibracket appliances. In their study, Ahn et al. detected a reduced palatine bone plate and an enlarged buccal bone plate during retraction of the upper incisors using a multibracket appliance [17]. The present study also showed a similar bone reaction to the tooth movement created by the Invisalign aligners.

Overall, the present study showed an increase in bone in the sagittal direction in the mandible, as the bone increase on the lingual side was greater than the bone reduction on the buccal side. This effect was seen particularly clearly at the level of the mid of the root length. This suggests that either increased bone apposition was taking place in the traction zone on the inner side of the lingual bone, or additional bone apposition on the outer side of the lingual alveolar bone. Treatment appears in general to have a slight positive effect on bone remodeling.

With regard to the bone height, vertical bone loss was detected overall. The largest and most significant vertical loss of bone was seen in the vestibular mandible. The reduction in bone height in the direction of the orthodontic tooth movement is even stronger than the change of the bone thickness in orovestibular direction. Extensive movement in the labial direction should be avoided during treatment planning. In this case other methods of creating space like i.e. IER or extractions should be considered. This is of high importance in respect of orthodontic treatment outcome, since long-term prognosis of tooth movement is only favourable, if the teeth remain firmly embedded in their bony alveolar sockets. Otherwise occurring recessions and a poor periodontal support might jeopardizing the success of orthodontic treatment. Dehiscences and fenestrations were observed on the CBCT scans between T0 and T1 in several cases. The long-term sequelae of this type of alveolar bone loss are as yet unclear. It is also unclear whether this type of damage is permanent or whether neo-osteogenesis is possible after a few months.

Akyalcin et al. [19] noted an increase in the buccal bone thickness after a 3-month retention period. Wainwright [14] reported a slight increase during a 4-month retention period. Follow-up research on the patients included in the present study would be desirable in order to investigate the positive or negative long-term results of Aligner treatment.

When the effects of the treatment in the maxilla and mandible are compared with regard to all the subtopics in the present study, different types of reaction are evident. Highly significant differences between the maxilla and mandible were noted. This is consistent with the findings of previous publications [20]. Movement out of the bony alveolar compartment is associated with bone dehiscences and fenestrations [6]. The size of the symphysis is decisive for the potential movement of the lower incisors. The labiolingual diameter of the bone is smaller in this area than in the maxilla, resulting in a very thin covering layer of bone on the lower incisors. The limits of the bone are therefore reached more quickly during tooth movements in the mandible. Using CBCT measurements in women, Uysal et al. found that bone sizes in the symphysis were smaller than in men [21]. Ninety-three percent of the patients included in the present study were women.

Another reason for the significant buccal bone loss in the mandible might be that the mandibular incisors generally have a smaller root surface in comparison with the maxillary incisors. In the mandible, the orthodontic force is thus distributed to a smaller periodontal ligament surface. The concentrated pressure on the buccal cortical plate of the lower incisors may therefore be greater and might therefore lead to a more severe reduction in bone thickness vestibularly [22]. The adult crowding was usually earlier and more severe in the mandible than in the maxilla in the present group of patients. More extensive space-creating measures were therefore needed in an area with a smaller bone volume. The risk of dehiscences is accordingly greater.

With regard to the movement pattern, it was found that the pattern planned in the ClinCheck software program had a strong influence on the therapeutically induced bone change (Tables 4 and 5). Excessive expansion of the dental arch should therefore be avoided at all costs.

This retrospective study was based on CBCT scans. Other studies on measuring bone volume have only been carried out using two-dimensional imaging. Gribel et al. compared measurements obtained with CBCT and cephalometric radiography with direct measurements on dry skulls [23]. They found that dental volume tomography with a slice thickness of 0.3 mm was extremely accurate, with a mean deviation of 0.1 mm from direct measurements. For traditional measurements with cephalometric radiography, they observed a large difference of 5 mm and poor intraoperator reliability. Cephalometric radiography is therefore unsuitable for measuring bone thickness. Semenoff et al. investigated the suitability of digitized panoramic radiographs in comparison with bitewing radiographs and dental films for diagnosing alveolar bone resorption [24]. Bone losses can also only be assessed imprecisely on panoramic radiographs, as they were often overestimated [24]. Zachrisson and Alnaes used dental films to study the interdental bone height in patients who had undergone orthodontic treatment [10]. They drew attention to the problem of the absence of a third dimension on all conventional two-dimensional radiographs, making precise assessment of buccal and lingual bone changes impossible.

Only three-dimensional images allow precise assessment of the bone. Hatcher and Aboudara considered that CBCT imaging is indicated in order to measure the buccal bone thickness when expansion is being planned [25]. In a study by Timock et al., CBCT with a voxel size of 0.3 mm (field of view 13 mm) was found to be suitable for measuring the buccal bone height and bone thickness quantitatively with a high degree of precision [26].

Unfortunaly 93 % of the patients included in the present study were women. A study group with a more balanced sex ratio would be desirable. Nevertheless, the publication should help to show data of the context between treatment of adult crowding with aligner and the possible bone change. It would be desirable to get more additional data from other hospitals to this subject. Although CBCT appears to have many advantages, the operator always needs to consider its use carefully for reasons of radiation protection. Considering the ALARA (As Low As Reasonably Achievable) principle CBCT is not indicated as a routine method of imaging periodontal bone support. A CBCT may be indicated in selected cases of infra-bony defects, where clinical and conventional radiographic examinations do not provide the information needed for management. If a CBCT is needed Cook et al. recommend the use of shorter scans and a reduced effective radiation dose for measurements of the buccal alveolar bone height and thickness [27]. Prospective RCTs (randomized controlled trials) would be interesting but could currently not be performed in accordance with the ALARA principle.

## Conclusions

Overall, Invisalign treatment for adult crowding with IER showed a dependence of the type of tooth movement and change of bone thickness. Bone changes in the direction of the tooth movement. It appears that the vestibular bone lamella in the mandible is decisive in determining the extent of treatment and the method used to relieve adult crowding, at least in adult female patients. This might help defining the correct indication for different methods of creating space like i.e. IER or extractions.

## Abbreviations

CBCT: Cone beam computed tomography scans
IER: Interproximal enamel reduction
